# Involvement of the genicular branches in cystic adventitial disease of the popliteal artery as a possible marker of unfavourable early clinical outcome: a case report

**DOI:** 10.1186/1752-1947-4-91

**Published:** 2010-03-18

**Authors:** Efthymios A Ypsilantis, Paul V Tisi

**Affiliations:** 1 Conquest Hospital, Saint Leonards-on-Sea, East Sussex, UK; 2 Bedford Hospital South Wing, Kempston Road, Bedford, Beds, UK

## Abstract

**Introduction:**

Cystic adventitial disease of the popliteal artery is a rare cause of non-atheromatous claudication. It usually requires surgery to improve the distance walked by patients.

**Case presentation:**

We report the case of a 44-year-old Caucasian man with unilateral symptomatic popliteal cysts extending to his genicular branches and associated with multilevel stenosis of his anterior tibial artery. A surgical evacuation of the cysts successfully restored his arterial patency and led to an objective haemodynamic improvement but was associated with early recurrence of symptoms.

**Conclusion:**

We suggest that the involvement of the genicular branches in cystic adventitial disease of the popliteal artery is a possible indicator of extensive adventitial degeneration and unfavourable clinical prognosis.

## Introduction

Cystic adventitial disease (CAD) of the popliteal artery (PA) is a rare but well-recognized non-atheromatous cause of claudication. Since it was first described in 1954 [1], more than 200 cases have been reported, predominantly affecting middle-aged men from Europe, US and Japan.

Histopathological features of the disease are cystic collections of mucinous material containing varying combinations of mucopolysaccharides and mucoproteins within the adventitial layer of the artery. The cysts exert extrinsic pressure on the arterial lumen, which accounts for the clinical manifestations of chronic lower limb ischemia, mainly intermittent claudication, and limb pain with absent distal pulses. Its aetiology is uncertain, with theories arguing about the possible degenerative, embryonic or ganglionic nature of the disease [2].

Diagnosis is usually achieved with duplex ultrasound, computed tomography (CT), or magnetic resonance imaging (MRI). Various approaches of treatment have been described, including percutaneous cyst aspiration, open incision and cyst enucleation, endovascular stenting, excision of the cyst with autologous vein graft reconstruction, and bypass surgery [3].

We report the case of a patient with unilateral claudication secondary to multiple adventitial cysts of the popliteal artery with additional involvement of the genicular arteries.

## Case presentation

A 44-year-old Caucasian man who works as a personal trainer presented with a four week history of unilateral (right) leg claudication occurring at a distance of 150 meters and exacerbated by running. He had no significant personal medical history, smoked five cigarettes per day, and engaged in extreme sports and vigorous exercise. His body mass index (BMI) was normal, although he had been morbidly obese ten years prior to presentation.

**Figure 1:**
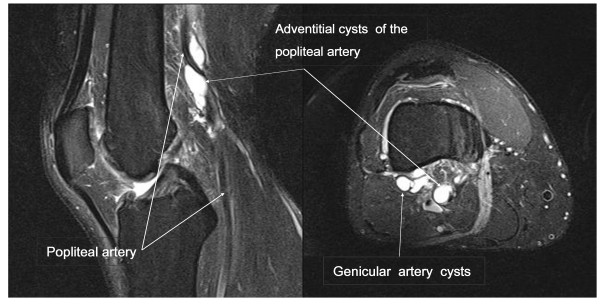
**T2-weighted image of our patient's magnetic resonance imaging scan**. The sagittal image on the left demonstrates one cyst in the posterior wall of the popliteal artery and a further anterior wall cyst causing stenosis. The axial image on the right shows the popliteal and genicular artery cysts. Lucent areas represent the arterial lumen.

On examination, all of our patient's lower limb pulses were palpable beside the dorsalis pedis on both his feet. His Doppler ankle-brachial pressure index (ABPI) was 1.09 on the affected side, with a 40 mmHg post-exercise pressure drop. Duplex ultrasound revealed three adventitial popliteal cysts, the largest measuring 3.4 cm (length) by 0.8 cm (diameter). A magnetic resonance angiogram confirmed a high-grade stenosis in his symptomatic proximal popliteal artery, as well as multiple stenoses in both his anterior tibial arteries but with a three-vessel bilateral run-off. An MRI scan of the affected popliteal fossa, performed to accurately assess the relations of the cysts to the surrounding structures and to exclude any other pathology, additionally showed involvement of his genicular arteries (Figure 1).

**Figure 2:**
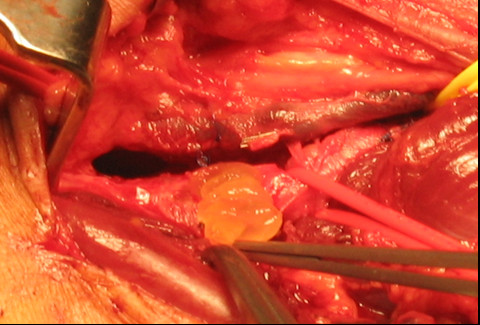
**Operative photograph showing typical contents of incised cyst on the posterior wall of the popliteal artery**.

Our patient underwent a surgical exploration of his popliteal artery under general anaesthesia through a posterior approach that allowed adequate exposure of the popliteal artery and cysts. Evacuation of all three cysts by longitudinal incision of his adventitia yielded yellow mucoid gelatinous material (Figure 2). The incised adventitia was sealed with bovine serum albumin or glutaraldehyde glue (BioGlue, Cryolife Europa, UK). He had an uneventful post-operative recovery, with immediate post-operative ABPI of 1.4. The yielded fluid contained acid mucin, which was demonstrated by positive mucicarmine and alcian blue staining.

He rapidly resumed normal activity after his discharge from our medical institution, to the extreme of cycling up to two miles daily four days post-operatively at his own initiative. However, his symptoms recurred four weeks later, with claudication of the same (right) limb occurring at a distance of more than half a mile and after exercise. A repeat Duplex scan demonstrated that his popliteal artery was widely patent with no evidence of recurrent stenoses. His ABPI was 1.36 with no pressure fall after exercise. In the absence of radiological evidence of popliteal artery stenosis, our patient was advised to avoid strenuous exercise, with a view to proceed to further imaging if symptoms recurred.

## Discussion

Although CAD of the popliteal artery was first described more than five decades ago, there is a growing published interest in the diagnosis and management of this rare condition [4-7]. Our case, along with the report of Crolla *et al. *[8] that focuses mainly on the diagnostic use of MRI in CAD, are the only reports describing the involvement of the adventitia of the genicular arteries. The early recurrence of symptoms in our patient, in the absence of any radiologically apparent luminal stenosis of the popliteal artery, raises the question of the potential significance of the involvement of genicular arteries in the disease outcome.

Multiple treatment options have been employed in the management of the disease. Despite reports of spontaneous resolution of symptoms [9], the majority of patients require surgery. Intravascular angioplasty and stenting have been described in recent case reports, but with conflicting and mostly unsuccessful results [10-12]. We proceeded to a less invasive incision and cyst enucleation, in favor of cyst excision and graft interposition, based on reported similar efficacy of this method [13-15].

Because of the rarity of the disease and the lack of large studies involving long follow-up examinations, the recurrence rate of previously treated CAD of the popliteal artery, or any associated risk factors, are not precisely known; it is, however, presumed to vary between six percent and ten percent, irrespective of the treatment method, with onset of recurrent symptoms between one and 21 months [13-15].

The proposed mechanism for an adventitial cyst to become symptomatic involves a sufficient increase of the pressure within the fluid-filled cyst during muscle exertion, thus resulting in haemodynamically significant endoluminal stenosis [6]. Communication of the cysts with the synovial structures in the knee have also been suggested, which accounts for a higher recurrence risk similar to Baker cysts after surgical excision [16]. In our case, this would be anatomically supported by the involvement of genicular branches. Also, the involvement of smaller-sized genicular arteries could imply that additional sub-radiological adventitial cysts might have affected the smaller arterial branches of the calf, thus causing recurrent claudication during strenuous exercise.

## Conclusion

CAD of the popliteal artery, although uncommon, should be considered in the differential diagnosis in young patients presenting with claudication, particularly if there are no risk factors for peripheral vascular disease. Our report raises the possibility that the extension of CAD to the genicular arteries could be a predictor of higher risk of recurrence, either as an indicator of cysts communicating with the knee synovium or as a marker of the involvement of smaller vessels elsewhere. Vascular surgeons should thus be encouraged to report similar cases in order to better identify risk factors of unsuccessful outcome based on larger series. Also, patients should be warned that they may not experience complete resolution of their symptoms despite objective evidence of surgical patency.

## Competing interests

The authors declare that they have no competing interests.

## Authors' contributions

EY performed the literature search and compiled data presented in this report. PT undertook the management of our patient from the time of his initial presentation to his surgery and follow-up examination. He also revised the manuscript draft. Both authors read and approved the final manuscript.

## Consent

Written informed consent was obtained from our patient for publication of this case report and any accompanying images. A copy of the written consent is available for review by the Editor-in-Chief of this journal.
